# Impact of 3-Year Period as a Factor on the Content of Biologically Valuable Substances in Seeds of White Lupin

**DOI:** 10.3390/plants11162087

**Published:** 2022-08-10

**Authors:** Ivana Tirdiľová, Alena Vollmannová, Silvia Čéryová, Peter Obtulovič, Július Árvay, Erika Zetochová

**Affiliations:** 1 AgroBioTech Research Center, Slovak University of Agriculture in Nitra, Trieda Andreja Hlinku 2, 949 76 Nitra, Slovakia; 2 Faculty of Biotechnology and Food Sciences, Slovak University of Agriculture in Nitra, Trieda Andreja Hlinku 2, 949 76 Nitra, Slovakia; 3 Faculty of Economics and Management, Slovak University of Agriculture in Nitra, Trieda Andreja Hlinku 2, 949 76 Nitra, Slovakia; 4 Gene Bank of the Slovak Republic, Research Institute of Plant Production, Bratislavská 2795/122, 921 01 Piešťany, Slovakia

**Keywords:** white lupin, total polyphenol content, antioxidant activity, DPPH, FRAP, ABTS, phenolic acids, flavonoids

## Abstract

White lupin seed is a unique legume rich in protein and fiber contents, as well as phytochemicals with health potential that contributes to a reduced risk of dyslipidemia, obesity and intestinal dysfunction. This study was focused on the effect of the year on the contents of caffeic acid, 4-hydroxybenzoic acid, trans-ferulic, trans-*p*-coumaric, quercetin, myricetin, kaempferol, apigenin and genistein, as well as the antioxidant activity and total polyphenols, of seeds of eleven varieties (*Lupine albus*). The contents of individual phenolic substances were determined by high-performance liquid chromatography–HPLC. The total content of polyphenols and the antioxidant activity were determined spectrophotometrically. The results show that the lowest contents of phenolic acids were found in the seeds from 2018. The caffeic acid and trans-ferulic acid were the most represented among all phenolic acids, during all 3 monitored years (2017, 2018, and 2019). Our results confirm the significant influence of the year of cultivation on the bioactive substances’ content in the seeds, and this can be potentially useful for the appropriate selection of locations for lupine growers in Slovakia, taking into account the climatic conditions of the given location. This study provides information about a legume that is underutilized in human nutrition, which may be a valuable source of bioactive substances.

## 1. Introduction

Lupin (lat. *Lupinus*) belongs to the Fabaceae (*Leguminosae*), a large family containing important food and feed crops [1]. There are more than 450 different species of lupins [2]. The most common species, with agricultural relevance, that is used for human consumption are *Lupinus angustifolius* (blue lupin or Australian sweet lupin), *Lupinus albus* (white lupin) and *Lupinus luteus* (yellow lupin) [3]. Seeds of lupin have been traditionally consumed in Mediterranean areas [4]. White lupin cultivation is divided into two main geographical areas: the Mediterranean basin and northern Africa, and northern and southern America [1].

Lupins are minor legume crops characterized by their high seed protein content, and they are cultivated worldwide across more than one million hectares, presenting a wide array of human uses [5,6,7]. Some scientists have mentioned the medicinal and cosmetic uses of lupin (probably Lupinus albus), and even Hippocrates (400–356 BC) advised this crop be included in human nutrition [8]. There have also been many references to using this crop for improving soil properties [9]. Lupins are grown mainly for their utilization as feed for livestock and as green fodder, but the interests of the food and medicinal industries have increased in the growing of lupin, and this crop is being more commonly used in human dietary habits [1]. The modern lifestyle is represented as one of the main causes of the abundance of chronic diseases, which have become a frequent problem in modern society. Factors such as smoking, increased alcohol intake, physical inactivity and insufficient diet are related to the development of diseases such as high blood pressure, diabetes, obesity and dyslipidaemias [10]. The insufficient intake of fiber as well as vitamins and minerals, and inappropriate food composition associated with a high energetic intake, contrtibute to the occurance of these diseases [10].

These diseases have been identified as metabolic risk factors for the incidence of cardiovascular diseases. Cardiovascular diseases are the main cause of death in the developed world. These diseases are estimated to account for 31% of all deaths [11]. In recent years, dietary guidelines have suggested the inclusion of legumes as part of a healthy diet to prevent or reduce the risk of chronic diseases [10]. Recently, lupin seeds have begun to be added as an ingredient in foods such as pastries, dairy products and fermented foods [12]. In vitro and in vivo studies have shown that the addition of lupin seed flour or lupin-based processed foods has reduced the risk of dyslipidemia, diabetes, obesity, hypertension, and dysfunction of the bowels [12,13].

The seeds of lupin are unique in their chemical composition and biological functions [14]. Lupin seeds are very rich in proteins and fibre, are almost free of starch and phytoestrogens and contain several essential amino acids, minerals, antioxidants and lipids rich in unsaturated fatty acids [15]. Lupin dishes could be an attractive alternative source of digestible proteins and energy [16]. Lupin has a high protein content (20–48%), a high fiber content and a low lipid content [17]. In addition to nutrients, lupin seeds contain significant amounts of phytochemicals with health-promoting potential, such as phenolic compounds, phytosterols and tocopherols [18]. Lupin flour is mainly used as a food ingredient in food products such as pasta, bread, cake, pizza, tofu, pies and noodles [16]. These foods, which are fortified with lupin, are considered functional because they have been shown to have a beneficial effect on at least one physiological function in an organism, and contribute to the improvement of the health state or reducing the risk of disease [14]. However, undesirable quinolizidine alkaloids are also present in lupin seeds. These toxic compounds are a major safety concern for lupin-based foods [19]. The seeds and vegetative organs of most species of lupins contain poisonous, hot-tasting quinolizidine alkaloids and non-protein amino acids. However, there are also so-called sweet lupins that do not contain these substances [19]. Various methods have been proposed to obtain sweet lupins. One process is the bacterial removal of the quinolizidine alkaloid, which, as far as is known, has never been implemented industrially. Another approach is to develop low-alkaloid varieties that do not require a removal process [20]. The seeds of newly bred cultivars of domesticated *Lupinus* species (e.g., *L. albus*, *L. angustifolius*, *L. luteus* and *L. mutabilis*) are poor in alkaloids but are also less resistant to disease and predator infestation [21].

The food and health industry’s interest in growing lupin has increased significantly, and lupin is increasingly being used in human nutrition. Of all the bioactive compounds present in lupin seeds, phenolic compounds are primarily responsible for the antioxidant capacity of seeds. These bioactive substances can reduce the risk of cardiovascular disease, reduce insulin resistance, and improve the lipid profile [22]. The reintroduction of crops with interesting chemical compositions is one way of the way we can change eating habits to improve the health of the population. In some regions, white lupin has a long tradition of cultivation, and it is a traditional high-quality crop [4,9,14].

Climate change, which is a very noteworthy problem for humanity today, as well as for other organisms on Earth [23], may have a significant impact on the health of bioactive compounds [24]. Climate change involves immediate and rapid changes in many important environmental parameters that regulate ecosystem dynamics [25]. This rapid change can cause direct and secondary physiological effects on plants, including changes in plant secondary metabolism [24]. Plant secondary metabolites generally refer to compounds that are essential for the plant’s interaction with the environment, and are also used in the medical industry. They also play an important role in defence mechanisms, acting as important signaling molecules for various environmental stresses, and thus play an important role in plants‘ adaptation to extreme environments. Different environmental factors, such as temperature, light, ultraviolet B radiation, tropospheric O_3_, salinity and soil water content, can influence the biosynthesis and accumulation of secondary metabolites in plants [25].

Currently, we are facing extreme weather fluctuations related to global climate change. We assume that these consequences of climate change may also significantly affect the contents of substances in crops grown in Slovakia, and thus also the biologically valuable components of white lupin seeds.

The aim of this study was to examine the effects of the climatic conditions in the Slovak Republic during the whole years and summers of 2017, 2018 and 2019 on the contents of biologically active substances in the seeds of 11 varieties white lupin (*Lupinus albus*).

## 2. Results and Discussion

### 2.1. The Total Polyphenol Content in White Lupin Seeds

Table 1 shows the values of the total polyphenol content (TPC) in the seeds of the studied white lupin varieties measured in 2017, 2018 and 2019.

In 2017, the highest TPC content was found in the Alban variety (10933 ± 638 mg GAE/kg). The lowest value was evaluated in the Astra variety (6346 ± 211 mg GAE/kg). The highest TPC value in 2018 was reached by the variety Astra (7768 ± 265 mg GAE/kg) and the lowest by the variety Nelly (4373 ± 254 mg GAE/kg). The Astra variety reached the highest value of TPC in 2019. The lowest content value in the same year was reached by the Alban variety. The range of TPC values in the seeds of the examined white lupin varieties in 2019 was 5790–8371 mg GAE/kg. Statistically significant differences (Kruskal–Wallis test) in TPC between years have been observed in the varieties Alban (2017/2019) (*p* < 0.01), Astra (2017/2019) (*p* < 0.01), R-933 (2018/2019) (*p* < 0.01), Nelly (2017/2018) (*p* < 0.01), Pop I (2017/2019) (*p* < 0.05), Los Palacios (2017/2018) (*p* < 0.01), Primorsky (2017/2018) (*p* < 0.01), Solnecny (2017/2018) (*p* < 0.01), Weibit (2017/2018) (*p* < 0.01) and Wtd (2017/2019) (*p* < 0.01).

The results of authors Sbihi et al. [26] are not consistent with ours, as their average values are significantly lower compared to our measured TPC values (1151 mg GAE/kg). A team of authors under Martínez-Villaluenga [27] compared the TPC values in three white lupin varieties, the results of which were again lower (2230 mg GAE/kg, 2530 mg GAE/kg and 1820 mg GAE/kg) than the polyphenol contents we measured in the white lupin seeds. On the contrary, the average values of four selected varieties of white lupin measured by Karamać et al. [28] are in agreement with the values in our research (3710 mg GAE/kg; 2120 mg GAE/kg; 2970 mg GAE/kg; 3640 mg GAE/kg). Hamouz et al. [29], in their several-year study, confirmed that altitude and temperature differences during the growing season significantly affect the total polyphenol contents of plants. Changes in temperature, precipitation and sun radiation during the monitored years 2017/2018/2019 in relation to the determined total content of polyphenols are shown in Table 2. Rameshrad et al. [30] assessed the proportion of bioactive components, which could vary depending on climatic conditions, harvest season, cultivation site, soil conditions and agronomic practice. They also concluded that higher intensities of solar radiation combined with drier conditions in locations where crops are grown may result in the increased production of phenolic compounds by crops. This phenomenon occurs due to the activation of the defense mechanism of plant cells against oxidative stress, for which reactive oxygen species are responsible [30,31]. Lima et al. [32] claimed in their work that plants grown in areas with low temperatures and a low intensity of sunlight produce a higher content of bioactive substances, which is caused by the reaction of their defense mechanism to stress factors. Shach et al. [33] surveyed how the lack of water in crops leads to the loss of plasma in the plant cell, which is a type of osmotic stress that significantly affects crop productivity and yield. The crop will thus increase its production of defensive (bioactive) substances as a reaction against unfavorable vegetation conditions. All these findings are based on one principle—the mobilization of the plant’s defense mechanism, and therefore also the formation of polyphenolic substances as part of it, in case of any stress.

From Table 2, it is obvious that the average highest content of polyphenols (7916.4 mg GAE/kg d.w.) was evaluated in 2017, where we found the average shortest length of sun radiation (613.6 min) and average lowest temperature (15.0 °C) during monitored years in the growing seasons of plants (April, May, June, July), using the weather station of the Research Institute of Plant Production in Piešťany. We assume that the parameters in 2017 had an impact on the plants, which triggered defense mechanisms against these unfavorable vegetation conditions, resulting in an increase in TPC in the samples. On the contrary, the improvement in the weather in 2018, with a slight increase in temperature (18.4 °C), length of sunshine (817.5 min) and precipitation (47.4 mm), was recorded, compared to 2017 and 2019. This could contribute to the decline in TPC in crops. Our results correspond with the above claims of the authors [32,33].

### 2.2. The Antioxidant Activity in White Lupin Seeds

Table 3 shows the values of antioxidant activity (AA) of the seeds of the examined white lupin varieties determined by three methods—DPPH (2,2-diphenyl-1-picrylhydrazyl), FRAP (ferric-reducing antioxidant power) and ABTS (2,2′-azinobis-(3-ethylbenzothiazoline-6-sulfonic acid))—during the three years 2017, 2018 and 2019.

Antioxidant activity in lupin was measured using the three methods FRAP, ABTS and DPPH. The highest antioxidant activity (DPPH method) was found in the variety Alban (25.6 ± 3.2 mmol TE/kg d.w.) in 2017. The highest antioxidant activity (FRAP method) was found in the variety Solnečnyj (4.33 ± 0.42 mmol TE/kg d.w.) in 2019. Using the ABTS method, we measured the highest antioxidant activity (11.9 ± 0.91 mmol TE/kg d.w.) in the Weibit variety in 2019.

Statistically significant differences (Kruskal–Wallis test) in AA values, determined by the DPPH method, in the seeds of individual varieties between the monitored years were confirmed in the following cases: 2017/2018 (*p* < 0.01) (Alban variety); 2017/2018 (*p* < 0.05) (Astra variety); 2018/2019 (*p* < 0.01) (Satmarean variety); 2017/2018 (*p* < 0.05) (Nelly variety); 2018/2019 (*p* < 0.01) (Los Palacios variety); 2017/2018 (*p* < 0.01) (Primorsky variety); 2017/2018 (*p* < 0.01) (Solnecny variety); 2017/2018 (*p* < 0.01) (Weibit variety). Statistically significant differences between the years were also confirmed in the AA values determined by the FRAP method: 2018/2019 (*p* < 0.01) (Alban variety); 2018/2019 (*p* < 0.01) (Astra variety); 2018/2019 (*p* < 0.01) (variety R-933); 2017/2019 (*p* < 0.01) (Satmarean variety); 2018/2019 (*p* < 0.01) (variety Pop I); 2018/2019 (*p* < 0.01) (Los Palacios variety); 2017/2019 (*p* < 0.05) (Primorsky variety); 2017/2019 (*p* < 0.05) (Solnecny variety); 2018/2019 (*p* < 0.01) (Weibit variety). Statistically significant year-on-year differences in AA values determined by the ABTS method were confirmed in varieties: 2018/2019 (*p* < 0.01) (Alban variety); 2018/2019 *(p* < 0.05) (Astra variety); 2018/2019 (*p* < 0.01) (variety R-933); 2018/2019 (*p* < 0.01) (Satmarean variety); 2018/2019 *(p* < 0.01) (Los Palacios variety); 2017/2019 (*p* < 0.01) (Primorsky variety); 2018/2019 (*p* < 0.01) (Solnecny variety); 2018/2019 (*p* < 0.01) (Weibit variety); 2018/2019 (*p* < 0.05) (Wtd variety).

Martínez-Villaluenga et al. [27] measured the average value of AA, using the DPPH method, as 47.9 mmol TE/kg, which does not correspond to the values of antioxidant activity shown in our results. Our measurement results range from 9.23 mmol TE/kg to 25.6 mmol TE/kg. Karamać et al. [28] measured the AA values using the ABTS method for four varieties of white lupin, with the following results: 11.2 mmol TE/kg; 5.3 mmol TE/kg; 8.5 mmol TE/kg; 12.3 mmol TE/kg. These correspond to our measured values. Using the same method, the AA of selected white lupin varieties was also determined by Alshikh et al. [34], whose final values ranged from 2.5 to 18.04 mmol TE/kg. The team of Bernaert et al. [35] found in their research that the individual phytochemicals present in the complex sample show different AA values, and at the same time react differently to the different agents used in the different methods of AA determination, which gives rise to differences in the antioxidant activity data determined by different methods. Our results correspond to this scientific statement.

In 2018, the highest average temperature (18.4 °C), the highest average amount of precipitation (47.4 mm) and the highest average length of sunshine (736 min) were recorded in our growing area during the growing season. These conditions were the most optimal for the cultivation of white lupine in the three monitored years, meaning the plants were not exposed to stress caused by weather conditions and forced to activate their defense system. Part of the defense mechanism of plants is the increased production of polyphenolic compounds with antioxidant activity.

The lowest average value of the antioxidant activity of lupine seeds was recorded this year. On the contrary, in 2017, the lowest average temperature (14 °C) and the lowest average length of sunshine (613.6 min) were recorded during the growing season. The year 2019, in contrast to the other two monitored years, was characterized by an extremely low average amount of precipitation during the growing season (3.6 mm). The average AA values in 2019 and 2017 were higher compared to 2018. This indicates the activation of the defense system of lupin plants due to stress conditions, and thus the increased production of antioxidant substances in lupin seeds. The results of our study indicate that climatic factors such as temperature, sunshine and precipitation also significantly influenced the contents of antioxidant components in the seeds of the examined varieties of white lupine in a given year.

### 2.3. Phenolic Acids in White Lupin Seeds

Table 4 presents the average values of phenolic acid contents measured in white lupin seeds during the years 2017, 2018 and 2019.

From the results (Table 4), we can infer that the values of the contents of the monitored phenolic acids in the seeds of the examined varieties of white lupin in the years 2017–2019 differed significantly. Similarly, via AA evaluation, the lowest average values of the monitored phenolic acid contents were found in 2018. In the cases of 4-hydroxybenzoic acid and caffeic acid, their contents were below the detection level in 2018. The average contents of caffeic acid and trans-ferulic acid were also the lowest in 2018 (415 mg/kg and 4.1 mg/kg, respectively). This confirms the role of these compounds in the plant’s defense system. This statement corresponds with those of Harborne et al. [36], who in their study described that phenolic compounds are synthesized during normal plant development, and their production is influenced by the plant’s response to environmental stress as a defense mechanism. However, it should be mentioned that the content of caffeic acid was also below the detection level in 2019, with the exception of the Satmarean variety. Variety is therefore likely to be another factor influencing the bioactive content of white lupin seeds. The authors Tiwari et al. [37] in their paper stated that variety is one of the most important factors with a significant effect on the total polyphenol content of the crop. The type of polyphenolic compound, especially its chemical structure, also plays an important role. However, our results indicate that climatic factors are another factor playing an important role in the formation of polyphenolic compounds in lupin seeds.

In the contents of phenolic acids in white lupin, we confirmed statistically significant (Kruskal–Wallis test) differences between the years, as follows. In the variety Wtd: 2019/2017 (*p* < 0.01) (4-hydroxybenzoic acid); 2018/2019 (*p* < 0.01), 2018/2017 (*p* < 0.01), 2017/2019 (*p* < 0.01) (caffeic acid); 2019/2018 (*p* < 0.01), 2019/2017 (*p* < 0.01), 2017/2018 (*p* < 0.01) (*trans*-ferulic acid). In the Weibit variety: 2017/2019 (*p* < 0.01) (4-hydroxybenzoic acid); 2017/2019 (*p* < 0.01), 2017/2018 (*p* < 0.01), 2018/2019 (*p* < 0.01) (caffeic acid); 2017/2018 (*p* < 0.01), 2017/2019 (*p* < 0.01), 2019/2018 (*p* < 0.01) (*trans*-ferulic acid). In the variety Solnecny: 2019/2017 (*p* < 0.01), 2019/2018 (*p* < 0.01), 2017/2018 (*p* < 0.01), (4-hydroxybenzoic acid); 2019/2018 (*p* < 0.01), 2019/2017 (*p* < 0.01), 2017/2018 (*p* < 0.01), (caffeic acid); 2019/2018 (*p* < 0.01), 2019/2017 (*p* < 0.01), 2017/2018 (*p* < 0.01), (*trans*-ferulic acid). In the variety Primorsky: 2019/2017 (*p* < 0.01) (4-hydroxybenzoic acid); 2017/2018 (*p* < 0.01), 2017/2019 (*p* < 0.01), 2019/2018 (*p* < 0.01) (caffeic acid); 2017/2018 (*p* < 0.01), 2017/2019 (*p* < 0.01), 2018/2019 (*p* < 0.01) (*trans*-ferulic acid). In Los Palacios: 2019/2017 (*p* < 0.01), 2018/2019 (*p* < 0.01), 2017/2018 (*p* < 0.01) (4-hydroxybenzoic acid); 2019/2017 (*p* < 0.01), 2019/2018 (*p* < 0.01), 2018/2017 (*p* < 0.01) (caffeic acid); 2019/2018 (*p* < 0.01), 2019/2017 (*p* < 0.01), 2017/2018 (*p* < 0.01) (*trans*-ferulic acid). In the Pop I variety: 2017/2019 (*p* < 0.01) (4-hydroxybenzoic acid); 2017/2019 (*p* < 0.01), 2017/2018 (*p* < 0.01), 2018/2019 (*p* < 0.01) (caffeic acid); 2017/2019 (*p* < 0.01), 2017/2018 (*p* < 0.01), 2018/2019 (*p* < 0.01) (*trans*-ferulic acid). In the variety Nelly: 2017/2019 (*p* < 0.01) (4-hydroxybenzoic acid); 2017/2019 (*p* < 0.01), 2017/2018 (*p* < 0.01), 2018/2019 (*p* < 0.01) (caffeic acid); 2017/2018 (*p* < 0.01), 2017/2019 (*p* < 0.01), 2019/2018 (*p* < 0.01) (*trans*-ferulic acid). In the Satmarean variety: 2017/2019 (*p* < 0.01) (4-hydroxybenzoic acid); 2019/2018 (*p* < 0.01), 2019/2017 (*p* < 0.01), 2017/2018 (*p* < 0.01) (caffeic acid); 2019/2018 (*p* < 0.01), 2019/2017 (*p* < 0.01), 2017/2018 (*p* < 0.01) (*trans*-ferulic acid). In the variety R-933: 2017/2019 (*p* < 0.01) (4-hydroxybenzoic acid); 2017/2019 (*p* < 0.01), 2017/2018 (*p* < 0.01), 2018/2019 (*p* < 0.01) (caffeic acid). In the Astra variety: 2017/2019 (*p* < 0.01) (4-hydroxybenzoic acid); 2017/2019 (*p* < 0.01), 2017/2018 (*p* < 0.01), 2018/2019 (*p* < 0.01) (caffeic acid); 2019/2018 (*p* < 0.01), 2019/2017 (*p* < 0.01), 2017/2018 (*p* < 0.01) (*trans*-ferulic acid). In the Alban variety: 2017/2019 (*p* < 0.01) (4-hydroxybenzoic acid); 2017/2018 (*p* < 0.01), 2017/2019 (*p* < 0.01), 2019/2018 (*p* < 0.01) (caffeic acid); 2019/2018 (*p* < 0.01), 2019/2017 (*p* < 0.01), 2017/2018 (*p* < 0.01) (*trans*-ferulic acid).

Legume seeds contain phenolic acids, which include derivatives of hydroxybenzoic acid and hydroxycinnamic acid. The main hydroxybenzoic acids are gallic, vanilla, syringic, salicylic, *p*-hydroxybenzoic, dihydroxybenzoic and 2,3,4-trihydroxybenzoic. Caffeic, sinapic, ferulic, *p*-coumaric and chlorogenic acids are hydroxycinnamic acids in legume seeds. Their concentrations depend on the composition of the legume seed (type and variety of legume) [38,39]. Ferchichi et al. [40] reported the contents of 4-hydroxybenzoic acid in *Lupinus luteus* to be 0.54–4.55 mg/kg d.w., in *Lupinus angustifolius* to be ND–0.08 mg/kg d.w. and in *Lupinus albus* to be 0.77–0.65 mg/kg d.w. The contents of trans-*p*-coumaric acid in *Lupinus luteus* ranged from 0.56 to 4.47 mg/kg d.w, and in *Lupinus angustifolius* 0.46–0.62 mg/kg d.w., while in *Lupinus albus* the values were undetectable, which corresponds to our results for 2018 and 2019. The measured contents of caffeic acid in *Lupinus luteus* ranged from ND to 0.1, and in *Lupinus angustifolius* ND–0.11 mg/kg d.w., while the values in *Lupinus albus* were below the detection limit, which does not correspond to our results. The contents of *trans* ferulic acid in *Lupinus luteus* ranged from 0.26 to 0.39 mg/kg d.w., in *Lupinus angustifolius* 1.1–1.47 mg/kg d.w., and in *Lupinus albus* 1.35–1.67 mg/kg d.w., which correlates with our results obtained by analyzing the Primorsky and Solnecny varieties in 2018 and the Pop I variety in 2019. Their study confirmed that the chemical composition and nutritional quality of lupin seeds vary significantly between species and varieties. These statements also correspond to our results [40].

### 2.4. Flavonoids in White Lupin Seeds

In our study, we also dealt with the influences in 2017, 2018 and 2019 on the contents of flavonoids in the monitored white lupin varieties (Table 5).

The highest measured concentration (25.861 ± 0.074 mg/kg d.w.) of flavonoids was of myricetin in 2019, in the Solnecny variety, and the lowest (0.648 ± 0.409) was genistein in the variety R-933.

In white lupin seeds, we recorded statistically significant differences (Kruskal–Wallis test) in the flavonoid contents in the following years. In the Wtd variety: 2017/2018 (*p* < 0.01), 2017/2019 (*p* < 0.01), 2019/2018 (*p* < 0.01) (myricetin); 2019/2017 (*p* < 0.01) (quercetin); 2019/2017 (*p* < 0.01) (apigenin); 2017/2018 (*p* < 0.01) (genistein). In the Weibit variety: 2017/2018 (*p* < 0.01), 2017/2019 (*p* < 0.01), 2019/2018 (myricetin); 2018/2017 (*p* < 0.01), 2018/2019 (*p* < 0.01) (apigenin); 2017/2018 (*p* < 0.01) (genistein). In the Solnecny variety: 2019/2018 (*p* < 0.01), 2019/2017 (*p* < 0.01), 2017/2018 (*p* < 0.01) (myricetin); 2019/2017 (*p* < 0.01) (quercetin); 2019/2017 (*p* < 0.01) (apigenin); 2017/2018 (*p* < 0.01) (genistein). In the Primorsky variety: 2017/2018 (*p* < 0.01), 2019/2018 (*p* < 0.01) (myricetin); 2019/2017 (*p* < 0.01), 2019/2018 (*p* < 0.01) (apigenin); 2017/2018 (*p* < 0.01) (genistein). In the Los Palacios variety: 2019/2018 (*p* < 0.01), 2019/2017 (*p* < 0.01), 2017/2018 (*p* < 0.01) (myricetin); 2019/2017 (*p* < 0.01) (quercetin); 2019/2017 (*p* < 0.01) (apigenin); 2017/2018 (*p* < 0.05) (genistein). In the Pop I variety: 2017/2019 (*p* < 0.01), 2017/2018 (*p* < 0.01), 2018/2019 (*p* < 0.01) (myricetin); 2017/2018 (*p* < 0.01) (genistein). In the variety Nelly: 2017/2019 (*p* < 0.01), 2017/2018 (*p* < 0.01), 2018/2019 (*p* < 0.01) (myricetin); 2017/2019 (*p* < 0.01) (apigenin); 2017/2018 (*p* < 0.01) (genistein). In Satmarean variety: 2017/2018 (*p* < 0.01), 2017/2019 (*p* < 0.01) (myricetin); 2017/2018 (*p* < 0.01) (genistein). In variety R-933: 2017/2018 (*p* < 0.01), 2017/2019 (*p* < 0.01), 2019/2018 (*p* < 0.01) (myricetin); 2019/2017 (*p* < 0.05) (apigenin); 2017/2018 (*p* < 0.01) (genistein). In the Astra variety: 2017/2018 (*p* < 0.01), 2017/2019 (*p* < 0.01), 2019/2018 (*p* < 0.01) (myricetin); 2017/2018 (*p* < 0.01) (genistein). In the Alban variety: 2017/2018 (*p* < 0.01), 2019/2018 (*p* < 0.01) (myricetin); 2019/2017 (*p* < 0.01) (apigenin); 2017/2018 *(p* < 0.01) (genistein).

We only recorded the contents of quercetin in all the varieties we monitored in 2017. The values ranged from 0.636 ± 0.401 mg/kg d.w. (Los Palacios) up to 1.081 ± 0.160 mg/kg d.w. (Alban/Astra). In 2018, we did not detect quercetin in any variety, and in 2019, quercetin was measured only in the varieties Los Palacios, Solnecny and Wtd. Corell et al. [41] stated that increases or decreases in the concentration of quercetin are influenced by environmental factors that act on the plant during vegetation. Ferchichia et al. [40] reported contents of keampferol in lupin in the range of ND–0.17 mg/kg d.w. The values of campferol measured by us in 2017 and 2018 were below the detection limit in all monitored varieties, which corresponds to their results, but in 2019 our results were higher, compared to the maximum values reported by the above authors.

Zhong et al. [42] found contents of genistein in lupin ranging from 22.30 ± 1.35 μg/g d.w. to 62.60 ± 5.19 μg/g d.w., which do not correspond to our values. In their study, they confirmed that the genotype, the environment, and the methods of processing and storage significantly affect the contents of phenolic compounds in legume seeds. Their results show that the contents of free and bound polyphenols in seeds were significantly affected by all genotypic and environmental factors. However, the observed changes in the contents of phenolic compounds were largely attributed to the influence of genotype. We assume that these reported factors may have significantly influenced the phenolic contents of our observed samples, and thus caused the differences in our measured values within the three observed years. Akbar [43] claimed that the presence of bioactive substances in lupin seeds varies according to the areas where the crops are grown. Ferchichia et al. [40] reported contents of apigenin in lupin in the range of ND–7.35 mg/kg d.w. These values correlate with our results. In 2018, apigenin was measured only in the varieties Satmarean, Primorsky and Weibit.

On the basis of the above results, we can also state that myricetin was the only flavonoid measured in all the years and varieties studied. The highest measured concentration (25.861 ± 0.074 mg/kg d.w.) of any flavonoid was that of myricetin, namely, in 2019 in the variety Solnechnyj.

Although we measured the myricetin contents in all the years we monitored, when comparing the average measured values of all the varieties in 2017 (15.495 mg/kg d.w.), 2018 (8.571 mg/kg d.w.) and 2019 (13.138 mg/kg d.w.), we can see that the lowest average content of this flavonoid was recorded in 2018. The year 2018 was the weakest in terms of the contents of the observed flavonoids in our samples. Winkel et al. [44] reported that phenolic substances are synthesized during normal plant development, and their production increases in response to environmental stress as a defense mechanism. We consider that the difference in our results in 2018 is due to the slight increases in the amounts of sunshine, precipitation and heat (i.e., the reduction in environmental stress on plants) during the growing season of the plants, compared to other years (Table 2).

The structural and chemical diversity of flavonoids depends on their different properties and functions in different plant species. Besides providing protection from ultraviolet radiation, these compounds also play an important role in protecting plants from pathogenic and herbivore attack [36].

The authors stated that the upper epidermal parts of leaf peels of Pisum sativum cv. argenteum, which are exposed to visible and UV light irradiation, showed higher levels of both anthocyanins and flavonol glycosides. This confirms the protective role of these metabolites induced by UV radiation. Thus, this study suggests that solar radiation plays an important role in determining the levels of flavonoids and phenolic acids in plant species [45].

In general, we can conclude that the contents of the studied phenolic compounds varied from sample to sample, thus confirming that environmental factors influence the flavonoid contents of white lupin seeds.

## 3. Materials and Methods

### 3.1. Plant Material and Desrition of Field Experiment

In this study, eleven varieties of white lupin (*Lupinus albus*) were the subject of survey. These varieties are stored in the Gene Bank of the Slovak Republic as part of the conservation of plant genetic resources (Table 6).

**Figure 1:**
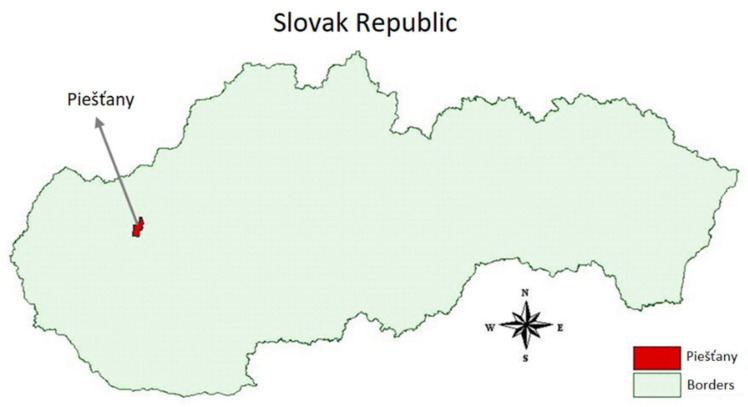
Map of the Slovak Republic with the town Piešt’any, which is the location of the Research Institute of Plant Production.

The town of Piešťany (Figure 1) is situated on the right bank of the river Váh, more precisely in the middle of the Považská promontory of the Danubian Lowland, at an altitude of 161 m. In terms of climate, this area is one of the warmest areas in Slovakia in terms of the number of sunny days per year.

The annual average temperature of this locality is 9.2 °C. The mildly dry and windy climate is typical of a lowland area, but the nearby mountains affect the direction, speed and wind flow. The annual average precipitation of this locality is 593 mm. Due to climatic conditions, this area is considered one of the most fertile areas in Slovakia. It was also confirmed by the fact that 63.5% of the soil belongs to the protected quality category. The soil type in experimental parcels at the Research Institute of Plant Production in Piešťany was degraded by chernozem, with the following mineral representation: very good content of K (284 mg/kg), medium content of Ca (4978 mg/kg), very high content of Mg (403 mg/kg), low content of P (44 mg/kg), high content of humus (3.33%), and neutral soil reactivity (6.9 pH/KCl). The dates of sowing in the field were as follows: 3 April 2017, 19 April 2018 and 12 April 2019. The dates of harvest were 31 July 2017, 30 July 2018 and 27 July 2019 (after the maturation of white lupin plants of each variety). No plant herbicides were applied to the white lupins during growth and hulled lupin grains were hand-separated, then dried at 45 °C (WTC Binder, Tuttlingen, Germany). When establishing field trials, crop rotation was performed in the same way, and legumes were always sown after cereals. The white lupin samples were collected in years 2017–2019 from the same 85.8 m^2^ area; the number of parcels was 11, and each of them were 5.2 m × 1.5 m.

### 3.2. Acquiring Meteorological Data

The automatic weather station Microstep-MIS is located in an experimental field in a fenced area. Parameters such as air temperature, ground temperature, soil temperature, air humidity, soil humidity, wind speed, wind direction, sun radiation, and precipitations are here measured. Once a month the data are transformed into an xls format file for easy further processing (Table 7).

### 3.3. Extract Preparation

All of the samples were dried before milling at 45 °C. The obtained crop seeds were homogenized using a laboratory mixer for a period of 30 s into a fine powder, and then extracts were prepared. Plant extracts were prepared according to the modified procedure of Rajurkar and Hande [46]. We weighed 5 g of the homogenized sample into extraction cartridges, which we extracted into 50 mL with 80% methanol for 8 h in a Twiselman extractor (BehrLabor-Technik, Düsseldorf, Germany). After this time, the obtained extract was filtered using a Munktell No. 1 filter. 390 paper (Munktell & Filtrak GmbH, Bärenstein, Germany) into 50 mL centrifuge tubes. Via this procedure, we prepared extracts from individual varieties of white lupin, which we used for the analysis of TPC, AA, phenolic acids and flavonoids.

### 3.4. HPLC-DAD Determination of Selected Phenolic Contents

All solvents and chemicals (including analytical standards) were of analytical or HPLC grade, and bought from Sigma-Aldrich (St. Louis, MO, USA). Prior to HPLC analysis, the extracts were filtered through a syringe filter, Q-Max (0.22 µm, 25 mm, PVDF) (Frisenette ApS, Knebel, Denmark). The contents of individual phenolics were determined using Agilent 1260 Infinity HPLC (Agilent Technologies GmbH, Wäldbronn, Germany) (the accuracy of the instruments is shown in Table 8) with a quaternary solvent manager coupled with a degasser (Cat. No. G1311B), a sampler manager (Cat. No. G1329B), a column manager (Cat. No. G1316A), and a diode array detector (Cat. No. G1315C) as previously reported by Lukšĭc et al. [47] and Germ et al. [48]. All HPLC analyses were performed on a Purospher^®^ reverse-phase C18 column (250 mm × 4 mm × 5 µm) (Merck KGaA, Darmstadt, Germany). The mobile phase consisted of gradient acetonitrile with phosphoric acid (pH = 3.5) (A) and 0.1% (*v/v*) phosphoric acid in deionized H2O (B). The gradient elution was as follows: 0–1 min isocratic elution (20% of A), 1–5 min linear gradient elution (20–25% of A), 5–15 min (25–30% of A), and 15–25 min (30–40% of A). The post-run was 3 min. The flow rate was 1 mL/min, and the injection volume was 5 µL. The column thermostat was set to 30 °C, and the samples were kept at 4 °C in the sampler manager. The spectral characteristics of the monitored analytes were scanned in the wavelength range 210–400 nm, while detection wavelengths were set at 320 nm (4-hydroxybenzoic acid, caffeic acid, trans-*p*-coumaric acid, trans-ferulic acid) and 372 nm (myricetin, quercetin, apigenin, kaempferol, genistein). Compounds were identified and quantified by comparing with the retention times of standard substances, and comparing the run of the spectral UV lines of the analytes. Data were collected and processed using the Agilent Open Lab Chem Station software for LC 3D systems (Agilent Technologies).

### 3.5. Total Polyphenols Contents

The total polyphenols contents were determined in methanol extracts via the method of Lachman et al. [49] with minor modifications, and by the spectrophotometric method, using a spectrophotometer and Folin–Ciocalteau reagent. The aliquot part of the prepared extract (2 cm^3^) was pipetted into a volumetric flask (50 cm^3^). The sample was diluted with distilled water, and 2.5 cm^3^ Folin–Ciocalteau reagent was added and thoroughly mixed. After 3 min of reacting, 7.5 cm^3^ (20%) of sodium carbonate was added and the flask was mixed again. Then, the distilled water was added up to the mark, and the mixed sample was left to react for 2 h (formation of blue complex). The calibration curve was prepared from the solution of gallic acid (5 µg·cm^−3^) and the blank was prepared. The absorbance of blue solutions against the control was measured at 765 nm using a Shimadzu UV-1800 (Kyoto, Japan). The results were calculated as milligram per kilogram of gallic acid equivalents in dry weight. The linearity range for this assay was determined as 0–150 μg/mL (R^2^  =  0.9948).

### 3.6. Determination of DPPH Radical Scavenging Activity

Antioxidant activity was determined by the method of Brand-Williams et al. [50] with some modifications, using DPPH radical (DPPH, Merck, Darmstadt, Germany). In total, 3.9 cm^3^ of the solution (DPPH) was pipetted into a cuvette, and then we determined the absorbance, where A0 means the initial concentration of the DPPH solution. Then 0.1 cm^3^ of the extract was added (according to individual plant species). The absorbance of the mixed solution was assessed by the spectrophotometer (Shimadzu UV–1800) in relation to the value of At (in time intervals; after 1 min to 10 min) at 515.6 nm. The antiradical activity of the tested extracts was calculated by using the formula for antioxidant activity. The Trolox (6-hydroxy 2,5,7,8-tetramethylchroman-2-carboxylic acid) provided in the kit was used as an antioxidant standard. A Trolox standard solution (concentration 0.1–2.0 mM) in 80% methanol was prepared and assayed under the same conditions. Free radical scavenging activity was expressed as millimoles of Trolox equivalents per kilogram dry weight on the basis of the standard curve (R^2^  =  0.9905). All samples were analyzed four times.

### 3.7. Determination of ABTS Radical Scavenging Activity

Antioxidant activity was determined by the protocol of Re et al. [51] using the ABTS radical (ABTS, Merck, Darmstadt, Germany). The acetate buffer (pH = 4.3) was prepared and then 3 mL of ABTS solution was pipetted into cuvettes. Then, 50 µL extracts from the tested crops were added. The absorbance was measured at 734 nm after 20 min (reduction in radical cation ABTS). The Trolox (6-hydroxy 2,5,7,8-tetramethylchroman-2-carboxylic acid) provided in the kit was used as an antioxidant standard. The Trolox standard solution (concentration 0.1–2.0 mM) in 80% methanol was prepared and assayed under the same conditions. The results were calculated as millimoles of Trolox per kilogram of the sample based on the calibration curve (R^2^  =  0.9573). All samples were analyzed four times.

### 3.8. FRAP assay for Antioxidant Activity Evaluation

Antioxidant activity was determined via a modified version of the method of Pedersen et al. [52] using 2,4,6-tris(2-pyridyl)-s-trianzine (TPTZ, Merck, Darmstadt, Germany). Acetate buffer (pH = 3.5) was prepared and aliquots of the solutions were mixed with a FRAP reagent followed by spectrophotometric measurement of the absorbance of the reaction mixture after incubation at 37 °C for 10 min at 593 nm against the blank. The FRAP assay has so far been widely used to directly test the total antioxidant potential of several foods and plant extracts, based on reduction in complexes of TPTZ with ferric chloride hexahydrate (FeCl_3_·6H_2_O), which are almost colorless. The solution will eventually turn slightly brownish, forming blue ferrous complexes after complete reduction. The Trolox (6-hydroxy 2,5,7,8-tetramethylchroman-2-carboxylic acid) provided in the kit was used as an antioxidant standard. The Trolox standard solution (concentration 0.1–2.0 mM) in 80% methanol was prepared and assayed under the same conditions. AA was expressed as millimoles of Trolox equivalents per kilogram dry weight on the basis of the standard curve (R^2^  =  0.9989). All samples were analyzed four times.

### 3.9. Statistical Analysis

Each analysis was performed four times and the results are presented as mean ± standard deviation. The Kruskal–Wallis test using the DUNN’S procedure for multiple pairwise comparisons was used to assess the statistical significance of the experimental data of the assessed total content of polyphenols, as well as the phenolic compounds and antioxidant activity, between the individual years 2017/2018/2019, at a level of significance *p* < 0.05.

These calculations were performed with MS Excel 2016 (Microsoft, Redmond, WA, USA) and XLSTAT 2014 [53] (Addinsoft, New York, NY, USA). The results refer to the comparison of the years 2017/2018/2019; therefore, for a better understanding and interpretation of the results, we recommend reading all tables line by line from left to right between the years 2017 and 2019.

## 4. Conclusions

We found that the contents of valuable components of white lupin seeds grown in Slovakia are influenced by changing weather factors, which are a consequence of global climate change. In the following period, we will focus on the sensitivity of individual varieties to the effects of climate change in the Slovak Republic. We are continuing our experiments with the cultivation of the same varieties of white lupin.

The interests of consumers, especially small growers and gardeners, in this legume in Slovakia have been steadily growing. Many such growers are also contacting the Slovak Gene Bank and looking for ways to get hold of the original varieties of this crop. The Slovak Gene Bank is therefore working on the possibility of establishing, in cooperation with small growers, on-farm cultivation, which consists in the long-term cultivation of old and regional varieties on farms, while maintaining the varietal purity and authenticity of the seed samples. However, the main and most important condition is to ensure suitable seeds for growers.

In cooperation with the Gene Bank, we can provide the consumer not only with sufficient seeds, but also with information that can be potentially useful for the appropriate selection of locations for white lupin growers in Slovakia, taking into account the climatic conditions of a given location. Additionlly, the information provided by our study on the health-promoting bioactive components contained in white lupin seeds may increase consumer awareness, and thus increase lupin consumption in Slovakia. In this way, we could also contribute to ensuring that the valuable genetic resources of our country’s plants are not lost from our fields.

## Figures and Tables

**Table 1:** Relation of the year of cultivation to polyphenols content in seeds of white lupin (mg GAE/kg d.w., means ± standard deviations of four independent determinations).

Variety	TPC (mg GAE/kg) d.w.		
	2017	2018	2019
Alban	10933 ± 638 ^a^	7289 ± 113 ^ab^	5790 ± 26 ^b^
Astra	6346 ± 211 ^b^	7768 ± 265 ^ab^	8371 ± 92 ^a^
R-933	6694 ± 81 ^ab^	6415 ± 167 ^b^	6880 ± 80 ^a^
Satmarean	7615 ± 36 ^a^	7563 ± 478 ^a^	7971 ± 273 ^a^
Nelly	9226 ± 928 ^a^	4373 ± 254 ^b^	6230 ± 203 ^ab^
Pop I	7000 ± 79 ^b^	7544 ± 287 ^ab^	7987 ± 484 ^a^
Los Palacios	7541 ± 122 ^a^	6476 ± 123 ^b^	7112 ± 346 ^ab^
Primorsky	7575 ± 243 ^a^	6369 ± 76 ^b^	6453 ± 203 ^ab^
Solnecny	7213 ± 50 ^a^	6221 ± 24 ^b^	6683 ± 143 ^ab^
Weibit	9604 ± 58 ^a^	6628 ± 41 ^b^	7283 ± 76 ^ab^
Wtd	7333 ± 88 ^a^	6577 ± 16 ^ab^	6165 ± 116 ^b^

Values represent mean of four repetitions ± standard deviation. Small letters (a, b) show statistically significant differences (*p* < 0.05) between years (2017/2018/2019) for each variety, as examined by multiple pairwise comparisons and Kruskal–Wallis tests. TPC—total polyphenols content expressed as milligrams of gallic acid equivalents (GAE) per kilogram of dry weight (d.w).

**Table 2:** Changes in temperature, precipitation and sun radiation during the monitored years 2017/2018/2019 in relation to the determined total contents of polyphenols.

	2017	2018	2019
**TPC (mg GAE/kg) d.w.**	7916.4	6665.6	6993.2
**Temperature °C**	15.0	18.4	17.5
**Rainfall/mm**	23.5	47.4	3.6
**Sunshine (min.)**	613.6	817.5	736.0

The table shows changes in temperature, precipitation and sunshine during the monitored years 2017/2018/2019 as average values for the monitored months April, May, June and July (growing season of plants). Total content of polyphenols shows the average values of all varieties in the monitored years 2017/2018/2019.

**Table 3:** Influence of year of cultivation on antioxidant activity of seeds of monitored white lupin varieties (mmol TE/kg d.w., means ± standard deviations of four independent determinations).

Variety	AA (mmol TE/kg) d.w.								
	DPPH			FRAP			ABTS		
	2017	2018	2019	2017	2018	2019	2017	2018	2019
Alban	25.6 ± 3.2 ^a^	11.9 ± 0.5 ^b^	13.4 ± 0.9 ^ab^	1.64 ± 0.03 ^ab^	1.59 ± 0.05 ^b^	2.77 ± 0.27 ^a^	7.29 ± 0.34 ^ab^	5.47 ± 0.26 ^b^	8.65 ± 0.42 ^a^
Astra	18.2 ± 1.0 ^a^	15.4 ± 0.9 ^b^	16.5 ± 1.0 ^ab^	1.52 ± 0.04 ^ab^	1.33 ± 0.01 ^b^	2.62 ± 0.11 ^a^	8.05 ± 0.41 ^ab^	7.74 ± 0.08 ^b^	8.46 ± 0.17 ^a^
R-933	15.1 ± 0.9 ^a^	15.4 ± 0.8 ^a^	16.7 ± 0.6 ^a^	1.67 ± 0.04 ^ab^	1.06 ± 0.11 ^b^	3.05 ± 0.04 ^a^	8.28 ± 0.09 ^ab^	7.60 ± 0.08 ^b^	9.07 ± 0.09 ^a^
Satmarean	14.7 ± 0.7 ^ab^	12.3 ± 1.1 ^b^	16.7 ± 0.9 ^a^	1.67 ± 0.04 ^b^	2.16 ± 0.15 ^ab^	3.13 ± 0.14 ^a^	7.48 ± 0.16 ^ab^	7.09 ± 0.17 ^b^	9.72 ± 0.15 ^a^
Nelly	22.9 ± 1.7 ^a^	11.1 ± 0.8 ^b^	12.0 ± 0.5 ^ab^	1.51 ± 0.05 ^a^	1.44 ± 0.21 ^a^	2.91 ± 0.06 ^a^	8.91 ± 0.63 ^a^	6.51 ± 0.26 ^a^	6.41 ± 0.05 ^a^
Pop I	14.3 ± 0.6 ^a^	14.1 ± 0.7 ^a^	15.2 ± 0.1 ^a^	1.61 ± 0.04 ^ab^	1.43 ± 0.05 ^b^	2.84 ± 0.19 ^a^	8.80 ± 0.21 ^a^	7.92 ± 0.38 ^a^	8.06 ± 0.28 ^a^
LosPalacios	15.4 ± 0.9 ^ab^	10.8 ± 0.6 ^b^	17.1 ± 0.9 ^a^	1.62 ± 0.04 ^ab^	1.34 ± 0.12 ^b^	3.89 ± 0.33 ^a^	8.11 ± 0.23 ^ab^	3.53 ± 0.14 ^b^	10.5 ± 0.1 ^a^
Primorsky	13.9 ± 0.4 ^a^	10.5 ± 0.7 ^b^	11.3 ± 0.5 ^ab^	1.58 ± 0.03 ^b^	1.72 ± 0.16 ^ab^	3.50 ± 0.12 ^a^	5.12 ± 0.10 ^b^	7.73 ± 0.07 ^ab^	11.0 ± 0.2 ^a^
Solnecny	22.0 ± 2.6 ^a^	12.4 ± 1.0 ^b^	19.0 ± 0.5 ^ab^	1.37 ± 0.23 ^b^	1.62 ± 0.39 ^ab^	4.33 ± 0.42 ^a^	7.87 ± 0.20 ^ab^	6.28 ± 0.13 ^b^	10.9 ± 0.8 ^a^
Weibit	24.2 ± 0.7 ^a^	12.1 ± 0.9 ^b^	13.0 ± 0.5 ^ab^	1.74 ± 0.09 ^ab^	1.24 ± 0.13 ^b^	2.53 ± 0.22 ^a^	8.78 ± 0.21 ^ab^	7.13 ± 0.34 ^b^	11.9 ± 0.9 ^a^
Wtd	9.23 ± 1.06 ^a^	10.4 ± 0.3 ^a^	10.0 ± 0.5 ^a^	1.33 ± 0.14 ^a^	1.41 ± 0.08 ^a^	2.93 ± 0.17 ^a^	7.63 ± 0.06 ^ab^	7.19 ± 0.84 ^b^	11.6 ± 0.6 ^a^

The values represent the means of four repetitions ± standard deviation. Small letters (a, b) show statistically significant differences (*p* < 0.05) between years (2017/2018/2019) for each variety, concerning the method (DPPH, FRAP and ABTS, respectively) as examined by multiple pairwise comparisons with a Kruskal–Wallis test. AA—antioxidant activity expressed as millimoles of Trolox equivalents (TE) per kilogram of dry weight. Antioxidant properties were determined spectrophotometrically by different methods using radicals 2,2-diphenyl-1-picrylhydrazyl (DPPH), 2,2′-azinobis-(3-ethylbenzothiazoline-6-sulfonic acid) (ABTS) and the ferric-reducing antioxidant power (FRAP).

**Table 4:** Influence of the year of cultivation of phenolic acids contents in monitored varieties of lupin (mg/kg d.w., means ± standard deviations of four independent determinations).

Variety	(mg/kg) d.w.					
	4-Hydroxybenzoic Acid			Caffeic Acid		
	2017	2018	2019	2017	2018	2019
Alban	13.130 ± 0.343 ^a^	ND	5.414 ± 0.361 ^b^	743.3 ± 4.1 ^a^	242.2 ± 0.4 ^c^	645.8 ± 1.0 ^b^
Astra	8.744 ± 0.160 ^a^	ND	2.395 ± 0.175 ^b^	522.1 ± 0.2 ^a^	473.3 ± 0.2 ^b^	303.0 ± 9.9 ^c^
R-933	8.811 ± 0.408 ^a^	ND	2.226 ± 0.071 ^b^	544.6 ± 1.1 ^a^	457.3 ± 0.8 ^b^	355.9 ± 16.2 ^c^
Satmarean	10.510 ± 0.405 ^a^	ND	2.886 ± 0.098 ^b^	523.6 ± 0.6 ^b^	477.2 ± 0.5 ^c^	573.8 ± 0.5 ^a^
Nelly	17.760 ± 0.483 ^a^	ND	2.589 ± 0.187 ^b^	766.2 ± 1.5 ^a^	330.5 ± 0.5 ^b^	157.0 ± 0.4 ^c^
Pop I	9.506 ± 0.408 ^a^	ND	6.071 ± 0.018 ^b^	507.2 ± 0.4 ^a^	452.3 ± 0.6 ^b^	151.1 ± 0.2 ^c^
LosPalacios	5.625 ± 0.401 ^b^	ND	22.620 ± 0.194 ^a^	463.3 ± 0.4 ^c^	501.5 ± 0.4 ^b^	734.6 ± 27.5 ^a^
Primorsky	5.989 ± 0.246 ^b^	ND	10.801 ± 0.055 ^a^	485.8 ± 0.3 ^a^	419.6 ± 0.9 ^c^	449.2 ± 2.3 ^b^
Solnecny	8.475 ± 0.167 ^b^	ND	28.490 ± 0.901 ^a^	497.3 ± 0.2 ^b^	311.5 ± 0.3 ^c^	896.1 ± 4.1 ^a^
Weibit	9.583 ± 0.317 ^a^	ND	6.628 ± 0.920 ^b^	580.2 ± 0.3 ^a^	418.8 ± 0.4 ^b^	370.5 ± 0.4 ^c^
Wtd	5.393 ± 0.163 ^b^	ND	10.020 ± 0.127 ^a^	442.9 ± 0.5 ^b^	489.9 ± 0.2 ^a^	405.7 ± 0.6 ^c^
**Variety**	**Trans-*p*-Coumaric Acid**			**Trans-Ferulic Acid**		
	**2017**	**2018**	**2019**	**2017**	**2018**	**2019**
Alban	6.440 ± 0.343 ^a^	ND	ND	11.801 ± 0.321 ^b^	4.381 ± 0.325 ^c^	19.341 ± 0.413 ^a^
Astra	3.439 ± 0.160 ^a^	ND	ND	8.744 ± 0.160 ^b^	7.418 ± 0.168 ^c^	10.941 ± 0.439 ^a^
R-933	3.285 ± 0.406 ^a^	ND	ND	5.177 ± 0.635 ^a^	4.789 ± 0.407 ^a^	4.471 ± 0.247 ^a^
Satmarean	2.466 ± 0.403 ^a^	ND	2.283 ± 0.032 ^a^	6.806 ± 0.403 ^b^	5.896 ± 0.401 ^c^	7.896 ± 0.116 ^a^
Nelly	5.623 ± 0.483 ^a^	ND	ND	9.717 ± 0.485 ^a^	3.538 ± 0.481 ^c^	7.249 ± 0.216 ^b^
Pop I	2.936 ± 0.408 ^a^	ND	ND	6.968 ± 0.635 ^a^	5.700 ± 0.408 ^b^	1.659 ± 0.055 ^c^
LosPalacios	3.326 ± 0.407 ^a^	ND	ND	7.630 ± 0.565 ^b^	2.844 ± 0.406 ^c^	11.601 ± 0.158 ^a^
Primorsky	2.673 ± 0.242 ^a^	ND	ND	6.484 ± 0.437 ^a^	1.792 ± 0.244 ^c^	3.181 ± 0.292 ^b^
Solnecny	3.370 ± 0.162 ^a^	ND	ND	7.632 ± 0.162 ^b^	1.387 ± 0.162 ^c^	19.031 ± 0.326 ^a^
Weibit	6.355 ± 0.243 ^a^	ND	ND	7.746 ± 0.243 ^a^	2.163 ± 0.241 ^c^	2.891 ± 0.048 ^b^
Wtd	3.895 ± 0.163 ^a^	ND	ND	5.692 ± 0.365 ^b^	2.633 ± 0.167 ^c^	7.493 ± 0.150 ^a^

The values represent the means of four repetitions ± standard deviation. Small letters (a, b, c) show statistically significant differences (*p* < 0.05) between years (2017/2018/2019) for each variety, concerning the phenolic acids as examined by multiple pairwise comparisons with a Kruskal–Wallis test. ND (not detected) = under the limit of detection (<LOD).

**Table 5:** Influence of the year of cultivation on flavonoid contents in tested varieties of lupin (mg/kg d.w., means ± standard deviations of four independent determinations).

Variety	(mg/kg) d.w.								
	Myricetin			Quercetin			Keampferol		
	2017	2018	2019	2017	2018	2019	2017	2018	2019
Alban	21.190 ± 0.324 ^a^	7.717 ± 0.328 ^b^	20.380 ± 0.744 ^a^	1.081 ± 0.331 ^a^	ND	ND	ND	ND	1.745 ± 0.275 ^a^
Astra	16.160 ± 0.165 ^a^	9.111 ± 0.476 ^c^	10.340 ± 0.079 ^b^	1.081 ± 0.160 ^a^	ND	ND	ND	ND	1.852 ± 0.009 ^a^
R-933	13.940 ± 0.406 ^a^	7.383 ± 0.407 ^c^	9.414 ± 0.087 ^b^	0.647 ± 0.408 ^a^	ND	ND	ND	ND	1.254 ± 0.045 ^a^
Satmarean	13.710 ± 0.403 ^a^	12.480 ± 0.401 ^b^	12.561 ± 0.101 ^b^	0.937 ± 0.450 ^a^	ND	ND	ND	ND	1.720 ± 0.149 ^a^
Nelly	20.620 ± 0.483 ^a^	8.157 ± 0.481 ^b^	4.167 ± 0.093 ^c^	0.789 ± 0.483 ^a^	ND	ND	ND	ND	1.151 ± 0.058 ^a^
Pop I	14.980 ± 0.408 ^a^	7.350 ± 0.410 ^b^	4.549 ± 0.048 ^c^	1.194 ± 0.438 ^a^	ND	ND	ND	ND	1.021 ± 0.009 ^a^
LosPalacios	11.150 ± 0.399 ^b^	7.906 ± 1.018 ^c^	23.761 ± 0.117 ^a^	0.636 ± 0.401 ^b^	ND	2.355 ± 0.049 ^a^	ND	ND	1.766 ± 0.109 ^a^
Primorsky	13.360 ± 0.242 ^a^	9.957 ± 0.244 ^b^	13.252 ± 0.108 ^a^	0.990 ± 0.256 ^a^	ND	ND	ND	ND	2.531 ± 0.292 ^a^
Solnecny	13.830 ± 0.167 ^b^	9.662 ± 0.865 ^c^	25.861 ± 0.074 ^a^	0.991 ± 0.162 ^b^	ND	2.614 ± 0.021 ^a^	ND	ND	2.746 ± 0.167 ^a^
Weibit	17.820 ± 0.247 ^a^	7.201 ± 0.166 ^c^	8.945 ± 0.258 ^b^	0.645 ± 0.247 ^a^	ND	ND	ND	ND	2.270 ± 0.051 ^a^
Wtd	13.680 ± 0.163 ^a^	7.352 ± 0.181 ^c^	11.291 ± 0.063 ^b^	0.799 ± 0.182 ^b^	ND	1.813 ± 0.137 ^a^	ND	ND	2.563 ± 0.322 ^a^
**Variety**	**Genistein**			**Apigenin**					
	**2017**	**2018**	**2019**	**2017**	**2018**	**2019**			
Alban	1.770 ± 0.321 ^a^	0.697 ± 0.325 ^b^	ND	1.868 ± 0.321 ^b^	ND	9.489 ± 0.268 ^a^			
Astra	1.670 ± 0.160 ^a^	0.896 ± 0.163 ^b^	ND	1.621 ± 0.201 ^a^	ND	1.763 ± 0.112 ^a^			
R-933	1.593 ± 0.406 ^a^	0.648 ± 0.409 ^b^	ND	1.095 ± 0.406 ^b^	ND	1.604 ± 0.011 ^a^			
Satmarean	1.578 ± 0.403 ^a^	0.688 ± 0.401 ^b^	ND	1.381 ± 0.403 ^a^	1.278 ± 0.401 ^a^	1.393 ± 0.247 ^a^			
Nelly	1.776 ± 0.483 ^a^	0.835 ± 0.483 ^b^	ND	2.614 ± 0.485 ^a^	ND	1.061 ± 0.035 ^b^			
Pop I	1.593 ± 0.406 ^a^	0.700 ± 0.408 ^b^	ND	1.642 ± 0.424 ^a^	ND	1.464 ± 0.141 ^a^			
LosPalacios	1.467 ± 0.399 ^a^	0.796 ± 0.406 ^b^	ND	1.272 ± 0.399 ^b^	ND	6.880 ± 0.175 ^a^			
Primorsky	1.336 ± 0.246 ^a^	0.797 ± 0.244 ^b^	ND	1.368 ± 0.242 ^b^	1.493 ± 0.244 ^b^	1.992 ± 0.013 ^a^			
Solnecny	1.635 ± 0.167 ^a^	0.793 ± 0.162 ^b^	ND	1.933 ± 0.167 ^b^	ND	5.757 ± 0.097 ^a^			
Weibit	1.490 ± 0.243 ^a^	0.688 ± 0.241 ^b^	ND	1.291 ± 0.292 ^b^	2.950 ± 0.241 ^a^	1.613 ± 0.058 ^b^			
Wtd	1.498 ± 0.163 ^a^	0.894 ± 0.162 ^b^	ND	1.398 ± 0.163 ^b^	ND	4.257 ± 1.576 ^a^			

The values represent the mean of four repetitions ± standard deviation. Small letters (a, b, c) show statistically significant differences (*p* < 0.05) between years (2017/2018/2019) for each variety, concerning the flavonoids as examined by multiple pairwise comparisons (Kruskal–Wallis test). ND (not detected) = under the limit of detection (<LOD).

**Table 6:** The country of origin and the cultural regions of the investigated white lupin varieties.

Variety Name	Cultural Regions (Latitude/Longitude)	Country of Origin
Alban	Piešťany (48°3500800 N/17°4805600 E)	France
Astra	Piešťany (48°3500800 N/17°4805600 E)	Spain
R-933	Piešťany (48°3500800 N/17°4805600 E)	Poland
Satmarean	Piešťany (48°3500800 N/17°4805600 E)	Romania
Nelly	Piešťany (48°3500800 N/17°4805600 E)	Hungary
Pop I	Piešťany (48°3500800 N/17°4805600 E)	Poland
Los Palacios	Piešťany (48°3500800 N/17°4805600 E)	Spain
Primorsky	Piešťany (48°3500800 N/17°4805600 E)	Russia
Solnecny	Piešťany (48°3500800 N/17°4805600 E)	Ukraine
Weibit	Piešťany (48°3500800 N/17°4805600 E)	Germany
Wtd	Piešťany (48°3500800 N/17°4805600 E)	Poland

**Table 7:** Meteorological data during growing season of eleven varieties of white lupin within years 2017/2018/2019 in the Research Institute of Plant Production in Piešťany.

Month	Temperature °C			Rainfall/mm			Sunshine (min)		
	**2017**	**2018**	**2019**	**2017**	**2018**	**2019**	**2017**	**2018**	**2019**
**APRIL**	9.7	13.6	12.3	24.6	17.5	8.9	614.8	746.8	561.8
**MAY**	16.1	17.8	12.9	23.2	27.2	3.3	746.7	824.3	1162.7
**JUNE**	16.0	20.4	23.1	19.4	106.0	0.9	471.7	930.9	697.1
**JULY**	14.2	21.7	21.6	28.1	39.0	1.4	621.2	767.8	522.5

Changes of temperature, precipitation and sun radiation during the years 2017/2018/2019 as average values for the chosen months April, May, June and July (growing season of plants).

**Table 8:** The accuracy of the instruments.

Analytics	LOD (μg/mL)	LOQ (μg/mL)	Linearity (R^2^)
4-OH benzoic acid	0.89	2974	0.9998
caffeic acid	1.01	3.34	0.9994
trans *p*-coumaric acid	0.92	3.04	0.9999
trans-ferulic acid	0.99	3.28	0.9997
myricetin	1.09	3.6	0.9994
quercetin	0.99	3.28	0.9996
apigenin	1.12	3.7	0.9999
kaempferol	1.05	3.47	0.9999
genistein	0.97	3.2	0.9997

LOD—limit of detection, LOQ—limit of quantitation.

## Data Availability

Not applicable.
